# Orthogonalization of far-field detection in tapered optical fibers for depth-selective fiber photometry in brain tissue

**DOI:** 10.1063/5.0073594

**Published:** 2022-02-14

**Authors:** Marco Bianco, Marco Pisanello, Antonio Balena, Cinzia Montinaro, Filippo Pisano, Barbara Spagnolo, Bernardo L. Sabatini, Massimo De Vittorio, Ferruccio Pisanello

**Affiliations:** 1 Istituto Italiano di Tecnologia (IIT), Center for Biomolecular Nanotechnologies, Via Barsanti 14, Arnesano, 73010 Lecce, Italy; 2 Dipartimento di Ingegneria dell’Innovazione, Università del Salento, Via per Monteroni, 73100 Lecce, Italy; 3 Laboratoire Kastler Brossel, Sorbonne Université, CNRS, ENS-PSL Research University, Collège de France, Paris 75005, France; 4 Dipartimento di Scienze e Tecnologie Biologiche e Ambientali, Università del Salento, Via per Monteroni, 73100 Lecce, Italy; 5 Department of Neurobiology, Harvard Medical School, Howard Hughes Medical Institute, Boston, Massachusetts 02115, USA

## Abstract

The field of implantable optical neural interfaces has recently enabled the interrogation of neural circuitry with both cell-type specificity and spatial resolution in sub-cortical structures of the mouse brain. This generated the need to integrate multiple optical channels within the same implantable device, motivating the requirement of multiplexing and demultiplexing techniques. In this article, we present an orthogonalization method of the far-field space to introduce mode-division demultiplexing for collecting fluorescence from the implantable tapered optical fibers. This is achieved by exploiting the correlation between the transversal wavevector *k*_*t*_ of the guided light and the position of the fluorescent sources along the implant, an intrinsic property of the taper waveguide. On these bases, we define a basis of orthogonal vectors in the Fourier space, each of which is associated with a depth along the taper, to simultaneously detect and demultiplex the collected signal when the probe is implanted in fixed mouse brain tissue. Our approach complements the existing multiplexing techniques used in silicon-based photonics probes with the advantage of a significant simplification of the probe itself.

## I. INTRODUCTION

In recent years, the demand for multifunctional neural implants able to simultaneously stimulate and record neural activity is increasing.^1–5^ Indeed, the ability to optically interface with the brain through optogenetic techniques has stimulated the development of implantable devices able to perform spatially resolved interrogation of neural circuits, motivating attempts to integrate and multiplex several optical stimulation channels in a single implantable device.^6–10^ Solutions for this problem are based on integrated photonic circuits,^7^ micrometer-sized light emitting diodes (μLEDs),^11–13^ and multipoint-emitting tapered optical fibers (TFs).^14,15^ In parallel, the advent of genetically encoded fluorescent indicators of neural activity^16–20^ propels a new need in the field: detecting and multiplexing fluorescence signals collected from the scattering brain tissue with spatial resolution. Current methods to achieve this aim are mostly based on space-division multiplexing and time-division multiplexing (SDM and TDM, respectively) or on a combination of the two. Space-division multiplexing is commonly implemented by using optical fibers arrays to reach different regions of the brain and exploit a fiber bundle to monitor the collected fluorescence intensity.^21^ This can reach very high density, with implantable bundles composed by several hundreds of micrometric optical fibers that move in tissue along paths of minimum resistance to detect fluorescence in the tri-dimensional space.^22^ However, SDM requires the implantation of multiple waveguides and does not provide depth-resolution along the implant direction. An alternative method was proposed by Li *et al.*^23^ who employed a linear array of μLEDs coupled to an optical fiber; each element of the array is independently activated, and the resulting signal collected by the optical fiber is demultiplexed with time-division multiplexing.

An alternative, emerging approach for increasing the capacity of implantable optical systems for the collection of functional fluorescence consists in mode-division multiplexing (MDM), in which optical signals are conveyed on a single multimode fiber and separated according to their distribution across guided modes. This is typically employed in communication systems with interferometric-based methods,^24^ and it has been introduced in the field of optical neural interfaces with the development of implantable tapered optical fibers (TFs). A TF consists of a single optical fiber smoothly tapered along its axis.^25^ The modal properties of the taper can be exploited to make a specific subset of guided modes to exchange energy with the environment at specific taper sections,^26,27^ and collection volumes can be spatially restricted by realizing micro-apertures along metal-coated TFs.^9^ This makes the taper an intrinsic mode-division multiplexer of fluorescence signals generated around the implant, with tailorable detection volumes.^9^ The question on how to modally demultiplex these signals to monitor functional fluorescence with depth selectivity remains open, as previously reported methods rely on time-division demultiplexing.^9,28^

In this work, we propose an orthogonalization scheme of optical signals conveyed by microstructured TFs (μTFs) to identify the depth at which light is collected by the implantable device. The method is based on disentangling the modal components of the collected fluorescence signals using orthogonalized vector components in the Fourier space. The photonic properties of the narrowing waveguide imply that the diameter at which light enters the narrowing waveguide determines the transversal component of the wavevector of the modes it couples with. This allows the identification of a basis of independent far-field patterns that can be exploited to define a multi-dimensional space for demultiplexing collected fluorescence.

## II. RESULTS

**FIG. 1.**
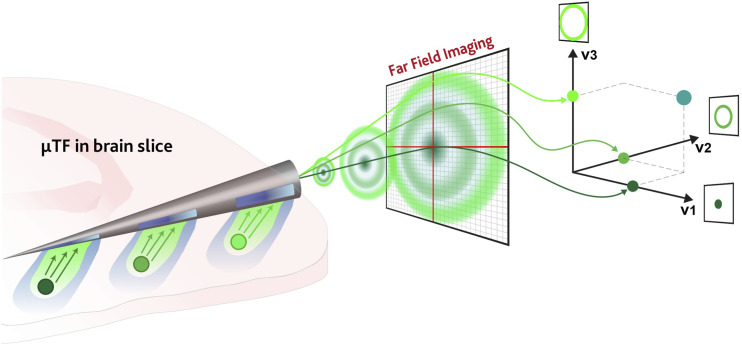
Schematic representation of the mode-division demultiplexing scheme proposed in this work. Photons entering the μTFs generate a far-field pattern that can be decomposed along specific basis vectors to identify the depth at which the fluorescence signal has been collected.

The working principle of the proposed method is described in Fig. 1. As photons entering the taper at different sections couple to different subsets of guided modes, the generated far-field image can be projected along the basis elements of a vector space whose definition enables the mode-division demultiplexing technique proposed in this work. Each basis vector can be associated with a microstructured aperture realized along the taper, allowing to identify the depth at which the fluorescence signal is collected.

In the following, we first show that it is possible to identify taper sections that can be mapped independently in the far-field space (Sec. II A) and engineer the position of detection points in μTFs thereof (Sec. II B) and then build a vector space to determine the depth at which the fluorescence signal enters the waveguide with pilot experiments in fixed mouse brain tissue (Sec. II C).

### A. High-resolution modal decomposition of point-like fluorescent sources in the far-field plane

The guided propagation in a TF collecting light generated by a fluorescent source can be modeled as a linear combination of linearly polarized modes.^29^ Depending on the diameter of the taper section at which fluorescence is coupled into the TF, the collected signal is guided through modes featuring different transversal components of the wavevector *k*_*t*_^30^ (a brief background is reported in the supplementary material, Note 1).

**FIG. 2.**
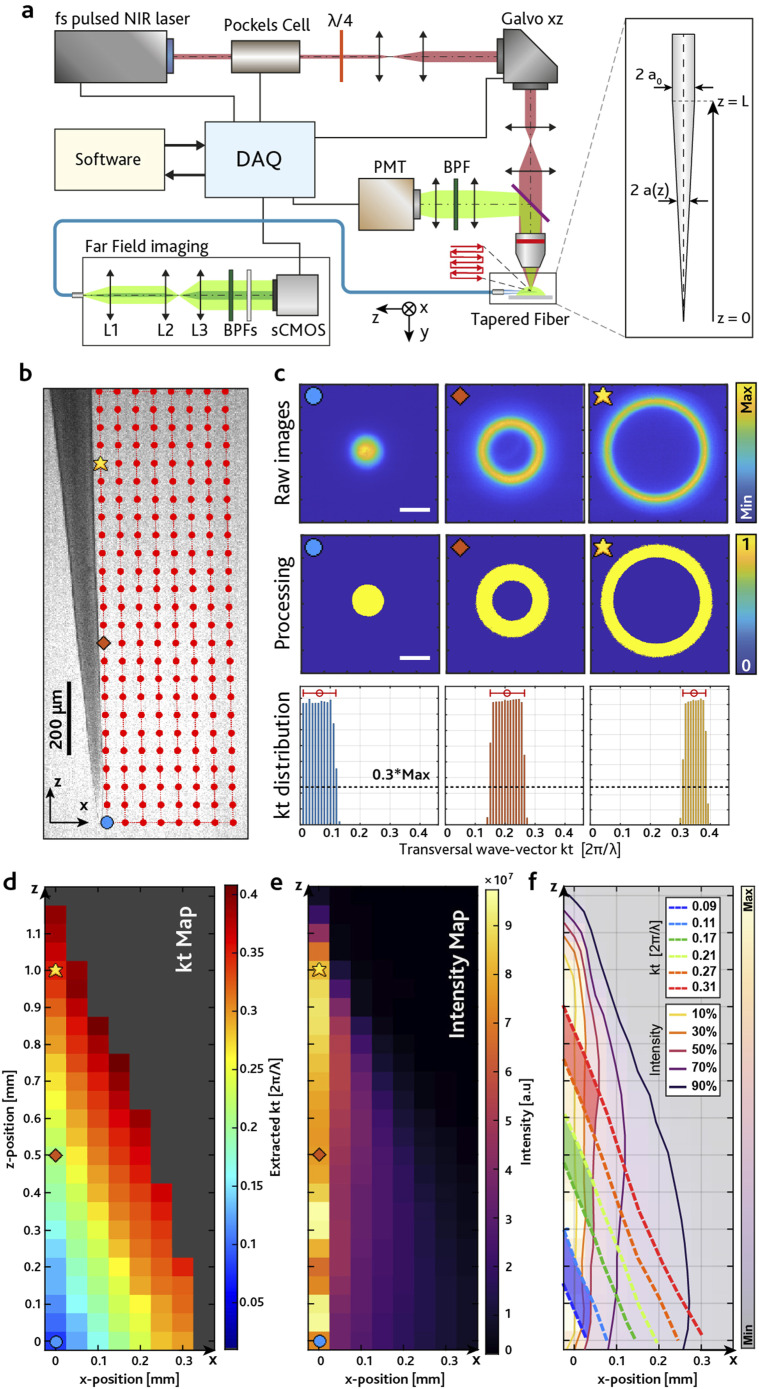
(a) Optical setup employed to excite fluorescence in a raster scan close to the TF. The fluorescence is collected by the TF and sent through an optical path to image the far-field of this signal on a sCMOS camera. Inset: schematic representation of a TF, showing the starting radius of the optical fiber *a*_0_ and the radius *a*(*z*) as a function of the *z* axis. *L* is the length of the tapered section. (b) Fluorescence image of a TF in PBS:fluorescein solution overlayed with red circles representing the points of the grid employed to acquire the *k*_*t*_(*x*, *z*) map of the TF. Blue circle, orange square, and yellow star represent three sample points, highlighted to show the subsequent steps of the processing. Scale bar represents 200 *μ*m. (c) Graphical description of the algorithm employed to extract the median *k*_*t*_ value from each image: (top) raw images acquired from the sCMOS camera when the light is collected by the TF in the corresponding points in panel (b). Scale bar represents 0.2·2*πλ*^−1^. [(c), center] Images of the same patterns after the segmentation. [(c), bottom] Histograms showing the *k*_*t*_ distribution collected in the same points. The red circle represents the extracted median *k*_*t*_, and the error bar shows minimum and maximum *k*_*t*_ of the histogram fitted with a top-hat function. (d) Representative wavevector map *k*_*t*_(*x*, *z*) extracted from the algorithm, obtained from a 0.37 NA fiber. Axes are concordant with panel (b). Points below a fixed frame intensity threshold are excluded and shown in gray. (e) Representative intensity map *I*(*x*, *z*) obtained from a 0.37 NA fiber. (f) Overlay of the isolines of maps in panels (d) and (e) to define spatial regions with non-overlapping ranges of *k*_*t*_ (highlighted in red, green, and blue).

To decipher how the position of a fluorescence source is linked to guided light, we have employed the setup shown in Fig. 2(a), described in detail in Ref. 9 and in Sec. IV. Briefly, a TF [sketched in the inset of Fig. 2(a)] is submerged in a fluorescent drop (30 *μ*M PBS:fluorescein) and the beam of a femtosecond-pulsed 920 nm laser is scanned in the [*x*, *z*] plane to simulate the presence of fluorescently stained cells beside the TF. Fluorescence collected by the TF propagates toward the flat distal facet whose far-field emission is imaged on a sCMOS camera. Light emerging from the distal fiber facet can be defined as a sum of plane waves^30^ with components *U*(*x*, *y*) propagating at different angles (*θ*_*x*_, *θ*_*y*_). Waves passing through lens L1 are focused on different points ***R***(*k*_*x*_, *k*_*y*_) on the sCMOS after an optical relay. $\left|R\right|$ is related to (*θ*_*x*_, *θ*_*y*_) and directly linked to *k*_*t*_,

$$k_{t}=\frac{2\pi }{\lambda }\sin\left[\tan^{-1}\left(\frac{f_{2}}{f_{1}f_{3}}\left|R\left(k_{x},k_{y}\right)\right|\right)\right],$$ (1)

where *f*_1_, *f*_2_, and *f*_3_ are, respectively, the focal lengths of lenses L1, L2, and L3 (a more detailed description of the far-field detection setup is reported in Sec. IV). A grid of *N* scanning points is defined in the [*x*, *z*] plane [Fig. 2(b)] to match the collection length of the TF (the light-sensitive region along the taper^9^). For each point in the grid, the acquired far-field image is segmented and processed by the algorithm summarized in Fig. 2(c), allowing to relate the collected average *k*_*t*_ to the position of the fluorescence source and assess the transversal wavevector spatial maps $k_{t}\left(x,z\right)$ [Fig. 2(d)].

The algorithm to extract the *k*_*t*_ average value starts with a stack of the collected far-field images *F*_*p*_, with *p* from 1 to *N*, resulting in rings of different diameters while the spot is moved across the points of the grid. Representative data are displayed in Fig. 2(c) (top). A gamma correction is then applied to increase the image contrast, and the images *F*_*p*_(*i*, *j*) are binarized, setting at 1 all pixels (*i*, *j*) receiving signal from the fiber and to 0 otherwise. Representative segmented images are shown in Fig. 2(c) (center). For each pixel (*i*, *j*) above threshold, the code retrieves the distance *R*_*p*_ from the center of the image (*i*_0_, *j*_0_),

$$R_{p}\left(i,j\right)=size_{pixel}\cdot \sqrt{{\left(i-i_{0}\right)}^{2}+{\left(j-j_{0}\right)}^{2}},$$ (2)

where *size*_*pixel*_ is the size of each square pixel of the camera. *R*_*p*_(*i*, *j*) values are then converted in wavevectors through Eq. (1). For each value of *p*, the *k*_*t*_ distribution is then evaluated to extract the median *k*_*t*_ values [see representative histograms in Fig. 2(c) (bottom)] plotted to obtain the *k*_*t*_(*x*, *z*) map. A representative *k*_*t*_(*x*, *z*) map is shown in Fig. 2(d) for a 0.37 NA TF with taper angle *ψ* ≅ 5° (a statistical analysis on n = 3 fibers for two different NAs is reported in the supplementary material, Fig. S1). Lower *k*_*t*_ values (corresponding to low order modes) are mostly collected by the fiber tip, and the detected *k*_*t*_ increases moving farther from the tip along the axial and radial directions. The *k*_*t*_(*x*, *z*) function is not injective in its [*x*, *z*] domain, since different excitation positions produce the same detected wavevector *k*_*t*_. However, evaluating *k*_*t*_(*x*, *z*) together with the collected intensity *I*(*x*, *z*) map [representative data in Fig. 2(e) and the supplementary material, Fig. S1] shows the possibility to define sections of the taper that can collect distinct *k*_*t*_ values. This is shown in Fig. 2(f), where isolines of both *k*_*t*_(*x*, *z*) and *I*(*x*, *z*) are overlayed: considering a detection threshold at 50% of the maximum intensity, non-overlapping ranges of *k*_*t*_ can be defined, highlighted by the red, green, and blue regions in Fig. 2(f). On these bases, in Secs. II B and II C, we first use the described mapping to engineer microstructured tapered optical fibers (μTF) for local fluorescence collection based on far-field detection, and then we propose a method to demultiplex the collected light based on non-overlapping *k*_*t*_ patterns in the far-field space, when the fluorescence is collected simultaneously from all the optical apertures.

### B. Detection of fluorescent sources with microstructured TFs

**FIG. 3.**
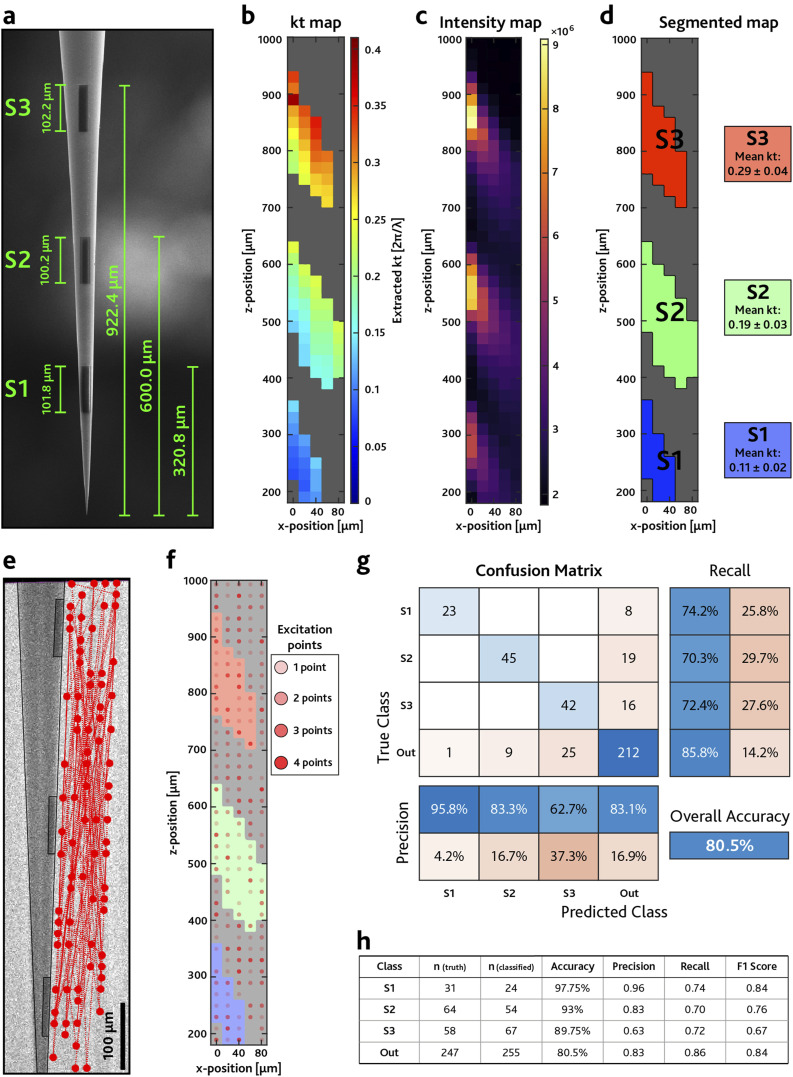
(a) SEM of the μTF with optical μSlots, showing the distance from the tip and the dimension of the optical apertures. (b) ***k***_***t***_ map extracted from the μTF with slots. It is possible to see three different collection regions near the position of the optical slots. ***k***_***t***_ values of the points in each region are averaged to obtain an estimation of the wavevectors collected by each aperture. (c) Intensity map of the light collected by the μSlots. It is possible to see three distinct collection lobes in the positions corresponding to the optical apertures. (d) Segmented ***k***_***t***_ map showing three ROIs with different wavevector intervals. (e) Fluorescence image of a μTF with μSlots in PBS:fluorescein solution. Scale bar represents 100 *μ*m. Overlayed red circles show ***N***_***R***_ = 100 randomly generated excitation points. The experiment was performed with ***N***_***R***_ = 400 points. In panel (e), we show 100 points for visualization sake. (f) Segmented ***k***_***t***_ map overlayed with ***N***_***R***_ = 400 randomly generated excitation points, represented by the red dots. From this map, it is possible to retrieve the True Class for the detection experiment. (g) Confusion matrix showing the results of the detection experiment, obtained by comparing the “True Class” and the “Predicted Class.” The overall accuracy of the experiment is 80.5%. (h) Table reporting different metrics for each class. The values are evaluated from the confusion matrix elements, according to Eq. (6).

On the base of the combined *k*_*t*_(*x*, *z*) and *I*(*x*, *z*) maps presented in Fig. 2(f), we fabricated microstructured TFs (μTFs) using Focused Ion Beam (FEI) lithography^9,31^ with light collecting micrometric slots (μSlots) positioned in specific taper sections to obtain non-overlapping *k*_*t*_ detection. This is shown in Figs. 3(a)–3(c), summarizing the results on a device featuring three μSlots defining spatial regions of interest (ROIs) S1–S3 along the fiber axis [scanning electron microscope images in Fig. 3(a) and close-ups in the supplementary material, Fig. S2]. Each ROI has the shape of a lobe extending for ∼200 *μ*m along the out-of-axis direction (higher resolution data are reported in the supplementary material, Fig. S2) and should be able to collect signal from tens of cells.^32^

In the following, we verify that light collected through the μSlots can be assigned univocally to a specific ROI and hence to define a basis to demultiplex the collected fluorescence.

To do so, we have implemented a blind source position detection experiment to simulate a stochastic distribution of neurons emitting functional fluorescence beside the implant. The wavevector map in Fig. 3(b) was segmented to assign a *k*_*t*_ value to each ROIs, obtained by averaging all the values in the corresponding region. Then, the *k*_*t*_ intervals for S1–S3 were defined by ${\bar{k}}_{t}\pm \sigma_{k_{t}}$ (mean ± standard deviation). Figure 3(d) shows the segmented map with the corresponding wavevector intervals for each ROI: ${\bar{k}}_{t,S1}=0.11\pm 0.02$, ${\bar{k}}_{t,S2}=0.19\pm 0.03$, and ${\bar{k}}_{t,S3}=0.29\pm 0.04$ (units of 2*πλ*^−1^).

For each of *N*_*R*_ = 400 randomly generated excitation positions [Fig. 3(e)], light was collected through the taper, and the algorithm in Fig. 2(c) was applied to assign the related *k*_*t*_ and classify it as detected by S1, S2, and S3 (referred to as Predicted Class). We then defined a true class by overlapping the *N*_*R*_ = 400 excitations to the segmented *k*_*t*_ maps [Fig. 3(f)] and compared the two in the Confusion Matrix (CM) in Fig. 3(g) to evaluate the performance of the classification model.^33^ The “Out” class was defined for excitations not being assigned to any ROI in the predicted or true classes.

In the CM in Fig. 3(g), most of the observations fall in the diagonal elements of the matrix, which represent the True Positives (TP) of the classification, meaning that the algorithm identifies correctly most of the randomly generated points (Overall Accuracy, OA = 80.5%). The first three elements of the last column (8, 19, 16) are points in S1, S2, and S3, respectively, that get classified as Out. Those elements are mostly associated with points at the boundaries of S1, S2, and S3 and are more likely to be misclassified by the code because in those boundary points the total frame intensity is close to the frame threshold. Similarly, the first three elements of the last row (1, 9, 25) represent points predicted to be in S1, S2, or S3 while had to be classified as Out. The other elements of the matrix, whose value is 0, represent the “cross-talk” between the slots, e.g., points falling in region S1 that get classified as S2 or S3. The table in Fig. 3(h) reports the metrics (defined in Sec. IV) used to evaluate the classifier output quality, showing good results in the experiment. Detection experiments were performed several times with similar results; the supplementary material, Fig. S3, shows details for other 10 experiments with *N*_*R*_ = 100 points each.

In Sec. II C, we extend this concept to achieve depth-resolved fluorescence collection from the brain tissue when photons are collected simultaneously from all the apertures. This is done by projecting the collected far-field patterns on a set of versors defined with the specific aim of demultiplexing the collected signal.

### C. Far-field detection in brain tissue

The experiments in Sec. II B demonstrate that the μSlots can be independently mapped in the far-field plane [*k*_*x*_, *k*_*y*_] with non-overlapping *k*_*t*_ intervals identified by averaging the transversal component of the wavevector detected in each of the grid points belonging to the different ROIs [Fig. 3(d)]. This lets us expect that the far-field patterns detected from each μSlot can be used as versors *v*_*n*_ to define a basis [*v*_1_, *v*_2_, *v*_3_] to demultiplex the collected fluorescence from the taper (see the supplementary material, Note 2, for details on the evaluation of the versors *v*_*n*_).

**FIG. 4.**
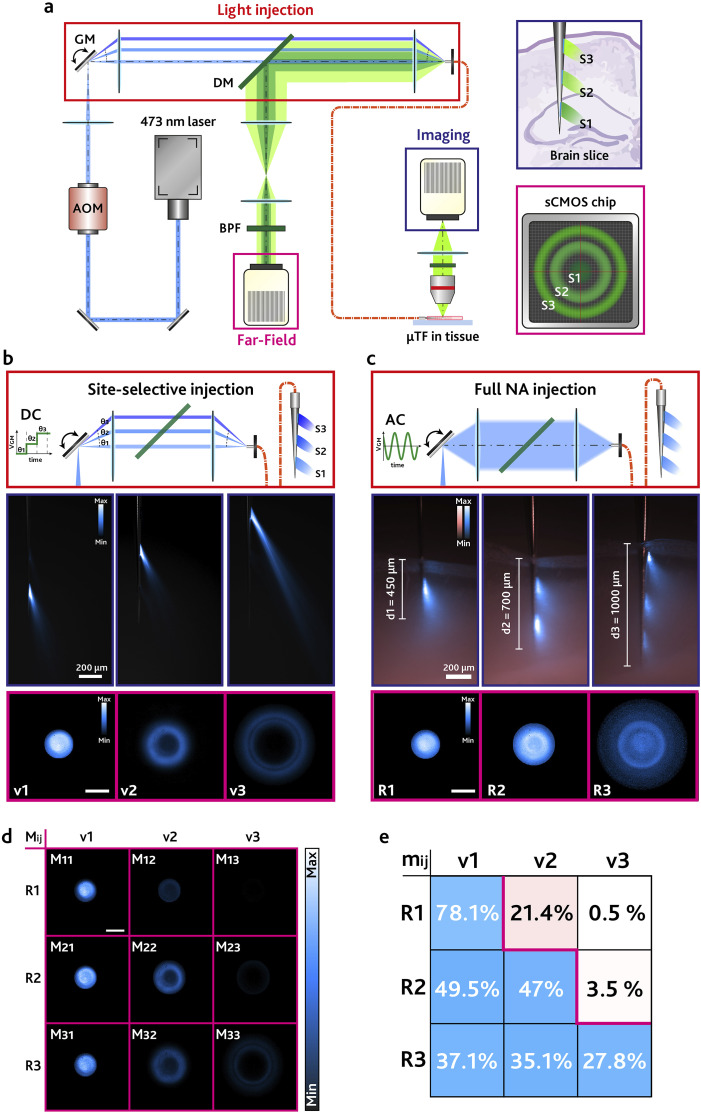
(a) Sketch of the setup employed for the injection of light in the μTF and the collection of the functional fluorescence. (b) (Top) Close-up sketches showing the site-selective injection method. (Center) Fluorescence images showing the light emission from each μSlot independently. (Bottom) Images ***v***_***n***_ detected by the sCMOS camera. Scale bar represents 0.2·2***πλ***^−1^. (c) (Top) Close-up sketches showing the full NA injection method. (Center) Fluorescence images showing the μTF being inserted in the cerebral cortex of a mouse brain slice. Pink field represents the fluorescence of the tissue generated by an LED source, and blue field represents the fluorescence generated in the tissue by the laser light outcoupled from the μSlots. Images show the implant depth (defined as ***d***_1_, ***d***_2_, ***d***_3_) in each configuration. (Bottom) Images ***R***_***i***_ detected by the sCMOS camera. Scale bar represents 0.2·2***πλ***^−1^. (d) Matrices ***M***_***ij***_ evaluated according to Eq. (3). Scale bar represents 0.2·2***πλ***^−1^. (e) Matrix ***m***_***ij***_ evaluated according to Eq. (5).

To identify a set of independent images, we have employed the setup in Fig. 4(a) to selectively activate each μSlot by delivering blue light in a PBS:fluorescein droplet and detected the resulting *v*_*n*_, with the obtained data displayed in Fig. 4(b). The same μTF was then gradually inserted in a 300 *μ*m-thick coronal mouse brain slice (*Thy*1-GCaMP6*s* GP4.12Dkim/J) expressing GCaMP6 fluorescence in the cerebral cortex and the hippocampus. Light was injected over the full NA of the TF to deliver light from all the μSlots simultaneously, and the far-field images *R*_*i*_ were acquired at the implant depths *d*_1_ ≅ 450 *µ*m, *d*_2_ ≅ 700 *µ*m, and *d*_3_ ≅ 1000 *µ*m [Fig. 4(c)]. As the μTF is inserted deeper in the tissue, light is progressively collected by all the μSlots simultaneously, as shown in Fig. 4(c). In this configuration, when the entire taper is implanted, the detected far-field pattern will have contributions from a growing number of μSlots, and it will appear broader with a larger distribution in the [*k*_*x*_, *k*_*y*_] space.

*R*_*i*_ images were then decomposed on the identified basis as follows. First, we employed the Hadamard product (element-wise product) between the detected patterns *R*_*i*_(*k*_*x*_, *k*_*y*_) and the basis versors *v*_*j*_(*k*_*x*_, *k*_*y*_) to obtain 9 images quantifying the overlap between the pattern detected by the μTF and the signal expected from each μSlot,

$$M_{ij}\left(k_{x},k_{y}\right)=R_{i}\left(k_{x},k_{y}\right)⊙v_{j}\left(k_{x},k_{y}\right),\;i,j=1,2,3.$$ (3)

Representative *M*_*ij*_ matrices are reported in Fig. 4(d), highlighting that most of the signal is extracted from the μSlots effectively implanted in the tissue. This is clearly seen in the case of matrices *M*_2*j*_: when the μTF is inserted with an implant depth *d*_2_, only S1 and S2 can excite fluorescence, and the detected signal is associated with matrices *M*_21_ and *M*_22_. Conversely, slot S3 outside of the brain slice cannot excite fluorescence, and *M*_23_ shows a negligible signal. When the μTF is implanted at a depth *d*_1_, most of the signal is found in *M*_11_, which is associated with S1. However, a smaller portion of the signal is associated with S2, since the light emitted by S2 can still excite fluorescence in the external layers of the cerebral cortex, as clearly seen in the corresponding fluorescence image in Fig. 4(c). This signal, collected by S2, is coupled to higher order modes with respect to S1 and observed in *M*_12_. Based on these considerations, we can define a dot product in the vector space to decompose *R*_*i*_ along the versors (details in the supplementary material, Note 2),

$$c_{ij}=\iint M_{ij}\left(k_{x},k_{y}\right)dk_{x}dk_{y},\;i,j=1,2,3.$$ (4)

The scalar *c*_*ij*_ is associated with each *M*_*ij*_ image by integrating the total intensity of the matrix, and it represents the fluorescence intensity signal from each μSlot. Finally, for each pattern *R*_*i*_, we evaluated the quantities *m*_*ij*_ by the ratio of *c*_*ij*_ and the sum of *c*_*ij*_ for each row,

$$m_{ij}=100\cdot \frac{c_{ij}}{\sum_{j}c_{ij}},\;i,j=1,2,3,$$ (5)

where *m*_*ij*_ represent the percentage of the total signal being detected by each μSlot in each implanting configuration [Fig. 4(e)]. As observed, most of the signal is collected by S1 when the implant depth is *d*_1_, and for increasing implant depths, it redistributes roughly evenly across the implanted apertures.

## III. DISCUSSION AND CONCLUSION

The rise of optogenetics and fiber photometry has generated a demand for conveying multiple optical channels in the same implantable device. Together with spatial and wavelength multiplexing, time-division multiplexing has been applied in multiple fiber photometry works, aiming at collecting functional fluorescence from multiple depths in brain tissue with a single implant^9,22,23^ (representative far-field time-division fluorescence collection data with μTFs are reported in the supplementary material, Fig. S4). Here, we take advantage of mode-division in multimode tapered optical fibers, exploiting the effect of the taper on the transversal component of the wavevector to implement an orthogonalization of detected far-field patterns to discriminate the depth at which the fluorescence signal is collected.

One crucial feature of TFs is that the detected photons are coupled to modes of different orders depending on the radius of the waveguide in each detection section. This allows us to decompose the fluorescence signal collected at different depths in the *k*_*t*_ space and to assign a portion of it to each realized μSlot by projecting the detected far-field patterns along the versor images *v*_*n*_ (Fig. 4), despite the scattering of the light induced by the brain tissue. One condition to avoid ambiguity in the assignment of the signal to each position is that the detection space defined by images *v*_*n*_ should be orthogonal, i.e., the *k*_*t*_ values detected by each aperture need to be non-overlapping. This is evaluated in the supplementary material, Fig. S5, where the results of the products *v*_*i*_ ⊙ *v*_*j*_ for *i*, *j* = 1, 2, 3 are reported, showing a negligible overlap between patterns. Indeed, the number of independent images *v*_*n*_ that can be defined along the fiber axis is a direct measurement of the spatial resolution of the method, since the overlapping patterns would result in ambiguity in the assignment of the signal to the μSlots. A strategy to increase the number of independent detection regions consists in reducing the dimension of the optical apertures: this would result in a smaller *k*_*t*_ range for each window/slot and in sharper and more separated versors *v*_*n*_. Although the segmentation algorithm described in Sec. II A allows us to extract a *k*_*t*_ value from a few above-threshold points in the far-field images (see the supplementary material, Fig. S4), reducing the dimension of the optical windows would diminish the total collected fluorescence signal; therefore, a compromise between mode-selectivity and intensity of the signal is needed. As modes available in an optical fiber increases with *NA* and core diameter of the waveguide, a method to further engineer the collection of guided modes could be the use of TFs with higher *NA* and larger core.

In conclusion, μTFs have the ability of probing fluorescence at multiple depths simultaneously, with multiplexing obtained by the intrinsic photonic properties of the narrowing waveguide and demultiplexing employed with simple far-field imaging. Despite fluorescence collection with implantable devices did not reach yet the spatial resolution obtained by devices for extracellular electrophysiology or for optogenetic stimulation, the ability of improving multiplexing capability of the technique can represent an important complement to reach this ambitious goal.

## IV. MATERIALS AND METHODS

### A. Fiber fabrication process

TFs were fabricated using step-index multimode fiber cords with core/cladding = 200/225 *μ*m with numerical apertures of 0.37 NA (Doric MPF 200/220/900-0.37) and 0.22 NA (Thorlabs FG200UEA). Starting from the cylindrical fiber, the tapered shape is obtained with the heat-and-pull method in which a segment of fiber is heated by a CO_2_ laser and gradually pulled. The pulling parameters were optimized in order to obtain the desired length, taper angle, and overall shape. After the fabrication, the samples are observed with a stereomicroscope to measure their geometrical properties. Samples were connectorized with a 1.25 mm ferrule using an epoxy resin, and the flat facet was then polished with lapping sheets.^34^ For experiments shown in Fig. 2 and the supplementary material, Fig. S1, we employed 0.37 NA TFs with an emission length *EL* ≅ 1250 *µ*m and *ψ* = 5°, and 0.22 TFs with *EL* ≅ 1650 *µ*m and *ψ* = 3°.

Metal coated TFs were fabricated by thermally evaporating 200 nm of Al all around the surface of the TFs. To obtain a uniform coating of the surface, the fibers are rotated with a stepper motor during the thermal evaporation process. The optical slots of μTFs were realized with focused ion beam milling (FEI Helios Nanolab 600i Dual Beam) by selectively removing the Al coating at specific sections of the fiber. In order to remove the Al without damaging the underlying glass, the process was supervised via simultaneous SEM imaging.^31^ For each TF, we fabricated three slot apertures along the TF axis, with dimensions of 100 × 20 μm^2^, at taper diameters of *a*_1_ = 30 *µ*m, *a*_2_ = 50 *µ*m, and *a*_3_ = 80 *µ*m for, respectively, S1, S2, and S3.

### B. Optical setup for TFs and μTFs characterization

The optical setup employed for the collection of the *k*_*t*_ maps consists of a two-photon laser-scanning microscope to excite fluorescence near the TF and a far-field detection path to image the collected fluorescence.^28,35^ With reference to Fig. 2(a), the beam of the fs-pulsed near-infrared (NIR) laser (Coherent Chameleon Discovery) is directed through a Pockels cell (Conoptics 350-80-02) to control the power, and then the beam, raised by a periscope, passes through a quarter-wave plate (λ/4, Thorlabs AQWP05M-980) to obtain circular polarization. The beam is enlarged by a beam expander and relayed to a scan head composed of two galvanometric mirrors (GMs) (Galvo xz, Sutter) that allow the scanning of the beam in the [*x*, *z*] plane. The beam is reflected in the *y*-direction and expanded to fill the back-aperture of the objective (Olympus XLFluor 4×/340), mounted upright on the microscope body (Olympus BX-61). The fluorescence generated by the laser spot is detected in the epifluorescence configuration: the signal is recollected by the same objective and directed, using a dichroic mirror (Semrock FF665-Di02), toward a band-pass filter (BPF, Semrock FF01-520/70-25) and focused on a non-descanned photomultiplier tube (PMT, Hamamatsu H10770PA-40). The image acquisition is controlled via the software ScanImage (Vidrio Technologies), and the components of the setup (laser, Pockels cell, PMT) are controlled by the digital acquisition board (DAQ, National Instruments). The TF, mounted on a three-axis micromanipulator (Scientifica PatchStar), is connected to a patch cord with a ferrule-to-ferrule junction. The fluorescence generated in the drop of PBS:fluorescein (30 *μ*M) is collected by the TF and guided by the patch cord toward the detection path.

**FIG. 5.**
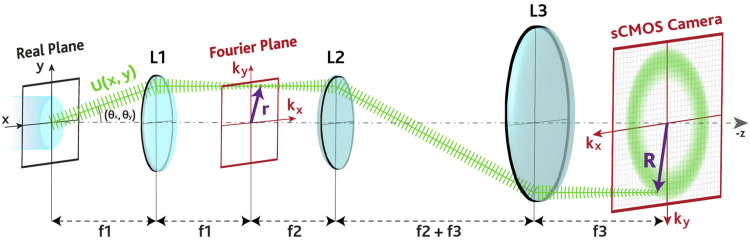
Representation of the far-field detection path. Plane waves re-emitted by the fiber and passing through L1 are separated and focused on different points ***r***(***k***_***x***_, ***k***_***y***_) at the back focal plane of L1, where the Fourier transform of the intensity of the fiber facet plane is found. The magnitude of ***r*** in the Fourier space [***k***_***x***_, ***k***_***y***_] is related to the angular distribution of the light out-coupled from the facet, related to the modal content. Lenses L2 and L3 magnify the far-field pattern to match the dimensions of the chip of the sCMOS camera. The sketch shows the optical path for a single plane wave being detected in the point ***R***; given the cylindrical symmetry of the TF, the far-field pattern on the sCMOS will also be symmetric along the radial direction.

Regarding the far-field detection system, sketched in Fig. 5, the light re-emitted by the patch cord is collected by lens L1 (ThorLabs AL4532-A, aspherical *f*_1_ = 32 mm) and focused on the back focal plane of L1. Lenses L2 (ThorLabs LA1301, *f*_2_ = 250 mm) and L3 (ThorLabs LA1050-N-BK7, *f*_3_ = 100 mm) magnify the far-field pattern to match the size of the chip of the scientific Complementary Metal–Oxide Semiconductor (sCMOS) camera (Hamamatsu C11440 Orca Flash 4.0, 2048 × 2048 pixel^2^, *size*_*pixel*_ = 6.5 *µ*m). Before being detected by the camera, the light is filtered by a NIR blocker and a band-pass filter (BPF) to detect only the light in the range of 500–550 nm wavelength.

### C. Far-field images acquisition and processing

The two-photon laser scanning microscope is controlled via the software ScanImage (Vidrio Technologies). In order to excite fluorescence nearby the TF, we used a stimulation protocol defined in Photostim, an API for ScanImage. The grids of stimulation points were defined with a custom MATLAB function. Far-field images were acquired with a sCMOS camera with 2048 × 2048 pixel^2^ and 16-bits grayscale depth in a TIFF format. To reduce the size of the images, the binning of the pixels was set to 4, obtaining square images with 512 × 512 pixel^2^ resolution. The size of each image is 512 kB, resulting in a 100 MB multi-page TIFF of raw images for each acquisition of *N* = 200 points. During the stimulation protocol, the image acquisition of the camera is triggered with the laser pulses by the DAQ board. The stimulation for each point of the grid consists in 450 ms laser excitation, followed by 50 ms of pause used as buffer to allow the repositioning of the galvo mirrors and the synchronization of the electronics. The exposure time on the camera was set accordingly to 500 ms. The same parameters were used when characterizing μSlots and during detection experiments. Data processing was performed on a workstation with Xeon E5-2630 v4 processor and 64 GB of random access memory, with MATLAB codes described in Fig. 2. A gamma correction (*γ* = 1.4) is applied to increase the image contrast, and then the images are binarized using a lower segmentation threshold set at the mean intensity detected by the sCMOS chip in dark conditions. Histograms extracted from the segmented images are divided by the radius of the corresponding circle in the [*k*_*x*_, *k*_*y*_] space to take into account that the signal arriving at larger ring radii is spread on more pixels. A histogram threshold is applied (0.3 · *Max*), and the histograms are fitted with a top-hat function to retrieve their minimum, median, and maximum *k*_*t*_ of the distribution for each image of the stack. In the *k*_*t*_(*x*, *z*) maps in Figs. 2(d) and 3(b), points below a fixed frame intensity threshold (evaluated as the total intensity detected by the sCMOS chip under dark conditions) are excluded and shown in gray. Intensity maps *I*(*x*, *z*) in Figs. 2(e) and 3(c) are evaluated by summing all the counts from each corresponding far-field image. A plot showing the computational time of the code as a function of the number of the far-field images *N* is reported in the supplementary material, Fig. S6.

### D. Definition of the excitation grids for the *k*_*t*_(*x*,*z*) and *I*(*x*,*z*) maps

The size of the excitation grids described in Figs. 2 and 3 matches the extension of the final characterization maps. Denser or sparser grids can be generated by varying the spacing between the points and the vertexes of the grid. For the characterization of bare TFs [Fig. 2(d) and supplementary material, Fig. S1], we defined a grid of 25 × 8 points, spaced 50 *μ*m from each other. This resulted in a [***x***, ***z***] domain composed of ***N*** = 200 points and a grid area ***A***_***G***_ ≅ 0.4 mm^2^, extending for 1250 *μ*m along the ***z*** axis, and 350 *μ*m along the ***x*** axis. Regarding the characterization of the μTFs with optical μSlots (Fig. 3), since μTFs collect less signal with respect to bare TFs, we realized denser grids with a shorter extension along the radial direction (***x*** axis). We defined a grid of 41 × 5 exciting points, spaced 20 *μ*m from each other, for a total on ***N*** = 205 scanning positions and a total area ***A***_***G***_ = 800 × 100 *µ*m^2^ = 0.08 mm^2^. For the close-up characterization of optical μSlots (see the supplementary material, Fig. S2), we defined grids extending for 250 × 100 *μ*m^2^ adjacent the optical aperture for a total area of ***A***_***G***_ = 0.025 mm^2^. To obtain higher resolution maps, we set the spacing between each point to 10 *μ*m for a total of ***N*** = 26 × 11 = 286 points.

### E. Metrics extracted from the detection experiments with μSlots

Figure 3(h) and the supplementary material, Fig. S3, report tables showing several figures of merit used to evaluate the quality of a classification model.

For each class, the quantities accuracy, precision, recall, and F1-score are defined as^33^

$$\begin{array}{c} Accuracy=\frac{TP+TN}{TP+TN+FP+FN},\\ Precision=\frac{TP}{TP+FP},\\ Recall=\frac{TP}{TP+FN},\\ F1-score=\frac{Precision\cdot Recall}{Precision+Recall}, \end{array}$$ (6)

where TP, TN, FP, and FN represent, respectively, the true positives, true negatives, false positives, and false negatives for each class.

The overall accuracy is referred to the overall detection experiment, rather than in the evaluation of a given class, and is defined as

$$OA=\frac{tr\left(CM\right)}{N_{R}}=\frac{\overset{n_{C}}{\underset{i=1}{\sum }}CM_{ii}}{N_{R}},$$ (7)

where CM is the confusion matrix, *n*_*C*_ is the number of classes, and *N*_*R*_ is the number of observations (the number of random excitation points in our case).

### F. Setup for the far-field detection in brain slices

In the far-field detection performed in brain slices, the light source was replaced by a continuous wave 473 nm laser coupled into the μTF with a patch cord. This allows for the excitation of fluorescence directly from the μTF, as in the case of typical fiber photometry experiments. We employed μTFs with three μSlots, with nominal dimensions of 100 × 20 μm^2^, as shown in Fig. 3(a). With reference to Fig. 4(a), the light of a 473 nm continuous wave laser (Laser Quantum Ciel) is power-controlled by an acousto-optic modulator (AOM, AA Opto-Electronic MT80-A1.5-VIS) and focused on the galvanometric mirror (GM, Cambridge Technology 6215H 5 mm) by a lens (ThorLabs LA1509-A). In the site-selective injection, the GM is fed with a constant voltage ***V***_***GM***_ to change its angular position. The light reflected by the GM is focused on the facet of the patch cord by a relay system composed of two lenses (ThorLabs AL50100-A and ThorLabs AL4532-A), with an angle ***θ***_***in***_ ∝ ***V***_***GM***_. This results in light being re-emitted only at specific sections of the μTF, depending on ***θ***_***in***_, and the emission of light only from a specific μSlot, allowing a time-division multiplexing detection.^9^ To obtain a full NA-like injection, the GM is driven by a sinusoidal voltage (>200 Hz) to fill the entire NA of the fiber by rapidly sweeping its angular span. This causes light to be injected in the TF with a multitude of angles, and be re-emitted by the entire active surface of the μTF, in a full NA-like fashion.^36^ Light coupled in the patch cord is re-emitted by the probe, generating fluorescence in the environment. The collected fluorescence is back-propagated and directed to the far-field imaging system described above through a dichroic mirror (DM, ThorLabs DMSP490L). The μTFs were implanted in the cortex of 300 *μ*m thick coronal mouse brain slices expressing GCaMP6 (mouse type: *Thy*1-GCaMP6*s* GP4.12Dkim/J). Blue light guided inside the patch cord and μTF generates an autofluorescence signal, which is detected by the sCMOS camera. Far-field patterns ***R***_***i***_ were collected in the brain tissue, and then the same acquisitions were performed with the μTF inserted in a drop of non-fluorescent solution (phosphate buffered saline) to acquire the autofluorescence pattern. The latter was used as background noise and subtracted from the ***R***_***i***_ images.^28^

## SUPPLEMENTARY MATERIAL

See the supplementary material for additional characterization of TFs and μTFs, details on the methods, and additional experiments.

## Data Availability

The data that support the findings of this study are available from the corresponding author upon reasonable request.
